# Phylogeography of *Rhodiola kirilowii* (Crassulaceae): A Story of Miocene Divergence and Quaternary Expansion

**DOI:** 10.1371/journal.pone.0112923

**Published:** 2014-11-12

**Authors:** Jian-Qiang Zhang, Shi-Yong Meng, Guang-Yuan Rao

**Affiliations:** College of Life Sciences, Peking University, Beijing, China; The National Orchid Conservation Center of China; The Orchid Conservation & Research Center of Shenzhen, China

## Abstract

The evolution and current distribution of the Sino-Tibetan flora have been greatly affected by historical geological events, such as the uplift of the Qinghai-Tibetan Plateau (QTP), and Quaternary climatic oscillations. *Rhodiola kirilowii*, a perennial herb with its distribution ranging from the southeastern QTP and the Hengduan Mountains (HM) to adjacent northern China and central Asia, provides an excellent model to examine and disentangle the effect of both geological orogeny and climatic oscillation on the evolutionary history of species with such distribution patterns. We here conducted a phylogeographic study using sequences of two chloroplast fragments (*trnL-F* and *trnS-G*) and internal transcribed spacers in 29 populations of *R. kirilowii*. A total of 25 plastid haplotypes and 12 ITS ribotypes were found. Molecular clock estimation revealed deep divergence between the central Asian populations and other populations from the HM and northern China; this split occurred ca. 2.84 million year ago. The majority of populations from the mountains of northern China were dominated by a single haplotype or ribotype, while populations of the HM harbored both high genetic diversity and high haplotype diversity. This distribution pattern indicates that HM was either a diversification center or a refugium for *R. kirilowii* during the Quaternary climatic oscillations. The present distribution of this species on mountains in northern China may have resulted from a rapid glacial population expansion from the HM. This expansion was confirmed by the mismatch distribution analysis and negative Tajima's *D* and Fu's *F*_S_ values, and was dated to ca. 168 thousand years ago. High genetic diversity and population differentiation in both plastid and ITS sequences were revealed; these imply restricted gene flow between populations. A distinct isolation-by-distance pattern was suggested by the Mantel test. Our results show that in old lineages, populations may harbour divergent genetic forms that are sufficient to maintain or even increase overall genetic diversity despite fragmentation and low within-population variation.

## Introduction

The evolution and genetic structure of extant species have been greatly affected by both geological history and climate oscillations [1], [2]. The Quaternary ice age played a critical role in shaping the current distributions and geographic structure of the genetic diversity of organisms in the North Hemisphere [1], [3]. Many phylogeographic studies of various species have shown that the spatial structure of genetic diversity is strongly associated with refugia during glaciations [4]. Although there was no major continental ice sheet in eastern Asia during the Quaternary [5], the continuing uplift of the Qinghai-Tibet Plateau (QTP) from the middle Tertiary has greatly changed the geology and topography of Eastern Asia. The QTP is the highest and largest plateau in the world [6], which harbors more than 12,000 species from 1500 genera [7]–[9]. Its uplifting has affected the distribution and genetic diversity of organisms found in this area [10]–[12]. Although phylogeographic studies on various plant groups from diverse geographic regions in China have increased greatly in recent decades, few of them have tried to disentangle the relative influences of both geographic history (e.g., the uplift of the QTP) and Pleistocene climatic oscillations in shaping the current distribution and genetic pattern of species in the QTP and adjacent area [13]–[15].

The Hengduan Mountains (HM), which are located at the southern and eastern edge of the QTP [16]–[18], have been considered the core area of the Himalayan biodiversity hotspot, which is of global importance [19]–[22]. It has long been thought that the severe alteration in the topology and climate of this area since the uplift of the QTP between the Miocene and the Quaternary triggered the origin of many plant taxa, both genera and species [23]–[25]. The Hengduan Mountains have also been suggested to have acted as a refugium for plants on the QTP and in the north temperate zone of eastern Asia during the Quaternary ice age [7]. More than ten plant species in the QTP and adjacent areas have been studied, focusing on their geographic patterns of genetic diversity. The results have confirmed that the Hengduan Mountains were an important refugium during the Quaternary ice age [26]–[31], although it is suspected that some species survived in ice-free areas on the plateau itself during the glaciation [32]–[34]. Many plant species distribute from the HM to northern China (e.g., *Rhodiola kirilowii* (Regel) Maxim., *Incarvillea sinensis* Lam., *Caragana jubata* (Pall.) Poir., *Hypecoum leptocarpum* Hook. f. et Thoms.), some of which could have originated and expanded northeast from the Hengduan Mountains [35], [36]. It is believed that northern China was not covered by an ice sheet during the Quaternary [1], [37], so determining whether species found in both southwestern China (QTP and adjacent area) and northern China experienced northerly expansion and southerly retreat during the Quaternary climate oscillation is of great interest. Although intensive phylogenetic and phylogeographic investigations of plants of the QTP and adjacent area have been conducted in recent decades [23], [29], [38]–[43], few studies have discussed the floristic relationship between the HM and the vast adjacent northern plain in northern China, which is dominated by homogeneous grassland [44]. Furthermore, most studies have focused on tree species, while herbaceous species have received much less attention [31], [42]. Thus, a phylogeographic study of an herbaceous species with a distribution that covers both the QTP and its adjacent areas and northern China in order to understand the evolutionary history of plants that exhibit similar distribution patterns is particularly valuable.

Habitat fragmentation could dramatically influence the demographic history of plant species [45], [46]. An increasing number of studies indicate that fragmentation could reduce genetic diversity and increase genetic divergence between populations because of restricted gene flow, inbreeding and genetic drift in isolated small populations [47]. On the other hand, global genetic variation could be maintained or even increased by a fragmented population, and allopatric speciation can occur due to genetic isolation [15], [48]. The genetic and ecological consequences of habitat fragmentation depend on other factors that affect genetic diversity: population size, gene flow and the timescale of fragmentation [49]. Thus, analysis of genetic divergence and gene flow between fragmented populations would help us to understand processes of speciation and extinction resulting from environmental changes. Besides, genetic diversity studies of naturally fragmented populations may reveal the consequences of population fragmentation over long periods of time and provide references for predicting consequences of fragmentation caused by human activity [50].

*Rhodiola* L. (Crassulaceae) consists of about 70 species mainly found at high- elevation in cold regions of the Northern Hemisphere, with the QTP and adjacent area representing its distribution center [51], [52]. A recent study [53] explored the origin and diversification history of *Rhodiola* and examined the biogeographic relationships between the QTP and other regions of the Northern Hemisphere. The results showed that the origin and diversification times of this group are largely correlated with the extensive uplifts of the Qinghai-Tibetan Plateau, and the ancestral area of *Rhodiola* is on the QTP, from where it dispersed to other regions of the Northern Hemisphere. *Rhodiola kirilowii*, one of the most widespread species of this genus, is distributed through the HM, northwest to the QTP platform and the Tianshan Mountains, and northeast to the plain of northern China [51]. Its distribution is naturally fragmented, especially in northern China, as the species often grows on alpine screes at the forest margin, usually 2000–5000 m in elevation. A range-wide phylogeographic analysis of *R. kirilowii* would be very helpful for exploring the floristic relationship between the QTP plus its neighboring area and the vast adjacent northeastern plain in northern China, and to reveal the consequences of natural habitat fragmentation. Our objectives included: (1) inferring the historical demography that could explain the current distribution pattern of *R. kirilowii* by examining the spatial pattern of plastid DNA (pDNA) and nrDNA ITS variation; (2) assessing the floristic relationship between the Hengduan Mountains and northern China; (3) revealing the consequences of natural habitat fragmentation on genetic diversity of *R. kirilowii*.

## Materials and Methods

### Ethics statement

No specific permits were required for the described locations in China because all researchers collecting the samples had introduction letters from College of Life Sciences, Peking University, Beijing. The field studies did not involve endangered or protected species. All locations of *R. kirilowii* populations sampled were shown in Table S1.

### Population sampling

Population sampling was conducted throughout the whole distribution range of *R. kirilowii* during the summers of 2009 to 2012, with the exact GPS coordinates and altitude shown in Table S1. Fresh leaves were collected from 29 populations and, with few exceptions, 10–12 individuals were sampled from each population. To avoid sampling closely related plants, the individuals sampled were at least 30 m apart. In total, 306 individuals of *R. kirilowii* were sampled and leaves were dried with silica gel. In addition, four individuals of *R. rosea* from different populations were sampled as outgroups.

### DNA extraction, PCR amplification, cloning and sequencing

DNA was extracted from silica-gel dried leaves with a Plant Genomic DNA Kit (TianGen Biotech, Beijing, China). The amplification primers used for ITS were ITS-1 and ITS-4 [52], for *trnL-F* were c and f [54] and for *trnS-G* were trnS and trnG [55]. Polymerase Chain Reaction (PCR) was performed in 20 µl reaction mixture volumes containing 2 µl 10× buffer, 0.5 µl of each primer, 0.4 µl of dNTP mixture, 1 U of Taq polymerase (TianGen Biotech, Beijing, China) and 1 µl template genomic DNA. The PCR cycling programs followed Liu et al. [56]. Direct sequencing was conducted for the pDNA fragments using the same primers as for the amplification. Most of the ITS samples were also directly sequenced, although a few of them were ligated onto pGEM-T Easy Vector using a Promega Kit (Promega Corporation, Madison, WI, USA). Plasmids containing the right insertion were chosen for sequencing reactions. All sequencing was conducted with a 3730 automatic DNA sequencer by Biomed Biotech, Beijing, China. Contigs were edited and assembled using ContigExpress (a component of Vector NTI Suite 6.0, InforMax). Sequence alignment was mostly done using ClustalW version 1.7 [57] and checked by eye in BioEdit version 7.0.1.

### Phylogeny and divergence time

Four accessions from *R. rosea* were chosen as the outgroup for the phylogenetic analysis based on previous phylogeny studies [58] and morphological classifications. Sequences from two pDNA regions (*trnL-F* and *trnS-G*) were concatenated into a matrix. Chloroplast haplotypes and ITS ribotypes were determined from both indels and nucleotide substitutions using DnaSP v5 [59], respectively. GenBank accession numbers of unique sequences of ITS and the two plastid fragments were KM459552–KM459594. Maximum parsimony and Bayesian inference analysis were implemented to reconstruct the phylogenetic relationships among haplotypes. Parsimony analysis was conducted using PAUP* version 4.0b10 [60]. In all parsimony analysis all characters were weighted equally and indels were treated as missing data. Heuristic searches with MULTREES and TBR branch swapping were conducted. Starting trees were constructed using 1000 replicates of random addition sequences. Support for individual nodes was assessed by bootstrapping [61]. For the bootstrap analysis PAUP* was set to run 1000 replicates with ten replicates of random addition sequences and NNI branch swapping, saving a maximum of 1000 trees of the random addition replicates. Nucleotide substitution model (TPM2uf+G for plastid data set, and SYM+G for the ITS data set) parameters were determined for Bayesian inference (BI) using the Akaike information criterion (AIC) in Modeltest version 3.7 [62], [63]. Bayesian inference was conducted using MrBayes version 3.2.1 (nst  = 6, rates  =  gamma for the plastid data set, nst  = 6, rates  =  gamma for the ITS data set) [64]. The search was started from a random tree using four Metropolis-coupled Markov chain Monte Carlo chains with 5,000,000 generations. The sampling rate of trees was every 1000 generations. To assess the convergence of two runs, the average standard deviation of split frequencies was used. The first ca. 10% of generations was discarded as the burn-in. The remaining data were used to construct a 50% majority rule consensus tree and the proportion of bifurcations found in this consensus tree was given as posterior clade probabilities (PP) to estimate the robustness of the BI trees.

In order to detect genealogical relationships among sequences with shallow genetic divergences, we constructed pDNA and ITS haplotype networks with NETWORK ver. 4.2.0.1 [65]. We hypothesized that both site mutations and indels evolved with equal likelihood and each indel was assumed to have originated independently of all other indels.

To infer the divergence time between lineages, we used the ITS data set to conduct a dating analysis with the BEAST software [66]. To examine the constancy of molecular evolution rate among lineages of the phylogenies, a likelihood ratio test was performed with PAUP version 4.0b10, in which likelihood scores of the trees with and without an enforced molecular clock were compared. Significance was calculated by comparing twice the difference in log likelihoods to a *χ* ^2^ distribution with *n*-2 degrees of freedom, where *n* is the number of taxa (haplotypes plus outgroups). A molecular clock could not be rejected for the ITS data (2logeLR  = 20.84, df  = 14, *P* = 0.11). We then used BEAST v.1.7.5 [66] to estimate divergence times and confidence intervals. We used GTR + G substitution models, a fixed molecular clock for the ITS data set, a constant population size coalescent tree prior and a UPGMA starting tree for both data sets. We sampled all parameters once every 2000 generations from 20,000,000 MCMC generations with a burn-in of 5,000,000 generations. We then used Tracer [66] to examine convergence of chains to the stationary distribution and the analysis was repeated before combining the two independent runs. As no fossils of *R. kirilowii* and its relatives were available, we used substitution rates to estimate divergence times. The substitution rate of nrITS in shrubs and herbs varies from 3.46×10^−9^ to 8.69×10^−9^ s s^−1^ yr^−1^ [67]. Considering the uncertainty of the rates, we used a normal distribution prior to cover these ranges with a 95% confidence interval, which is a mean of 6.075×10^−9^, and SD of 1.590×10^−9^.

### Population genetics

Haplotype diversity (*h*) and nucleotide diversity (*π*) for each population were calculated using DnaSP v5 [59]. Average gene diversity with a population (*H*_S_), total gene diversity (*H_T_*) and between population divergence (*G*_ST_, *N*_ST_) were estimated by the program PERMUT (available at www.pierroton.inra.fr/genetics/labo/Software/) with 1000 permutations tests. A higher *N*_ST_ than *G*_ST_ indicates the presence of phylogeographic structure, which means that closely related haplotypes will be found more often in the same area than more divergent haplotypes [68]. Measures of DNA divergence between the three geographically defined groups (i.e. the Hengduan Mountains, northern China, and central Asia) (*F*_ST_) were calculated with Arlequin version 3.5 [69], and the significance was tested using 10,000 permutations. To test the significance of isolation by distance between populations, the Mantel test with 1,000 random permutations on matrices of pair wise population *F*_ST_ values and the geographical distances was performed using Arlequin ver. 3.5 [69].

The spatial genetic pattern was examined by spatial analysis of molecular variance (SAMOVA) using SAMOVA v. 1.0 [70]. The program seeks the composition of a user-defined number (*K*) of groups of geographically adjacent populations that maximize the *F*_CT_ value (differences among groups of populations). We determined the value of *K* by repeatedly running the program with *K* = 2–10, and choosing the one which gave the maximum *F*_CT_ value. The amount of variation among populations within a region and within a population was calculated by the hierarchical analysis of molecular variance (AMOVA) framework carried out using Arlequin version 3.5 [69]. Significant differences were identified by a nonparametric permutation procedure with 1000 permutations.

We calculated Tajima's *D* and Fu's *F*s to identify signatures of demographic expansion of each clade recovered based on the plastid data set [71], [72]. If an expansion hypothesis was true, the *D* and *F*s statistic should have large negative values as a result of an excess of rare new mutations. We also conducted a mismatch distribution analysis [73], [74] to detect the population expansion of *R. kirilowii*. We pooled all haplotypes of each clade together without considering their frequencies because population structure has little effect on mismatch distribution [75]. We used 1000 parametric bootstrap replicates to test the fitness of observed mismatch distributions to the theoretical distribution under a sudden expansion model [74], [76], examining the sum of squared deviations (*SSD*) between observed and expected mismatch distributions, and the raggedness index (*HRag*) of Harpending [77]. When we identified that a group had experienced expansion, we used the parameter-value for the mode of the mismatch distribution (*τ*) to estimate time (in generations) since expansion with the equation *t* = *τ*/2*u* [74], [75]. Here, *u* was calculated as *u* = *µ*×*k*×*g*, where *µ* is the number of substitutions per site per year (s s^−1^ yr^−1^), *k* is the average sequence length in the present study and *g* is the generation time in years (age when first reproduction occurs). In the present study, our value for *k* was 1549 bp. The pDNA substitution rates for most angiosperm species have been estimated to be in the range 1−3×10^−9^ substitutions per site per year (s s^−1^ yr^−1^) [78]. As the two fragments we used are non-coding regions of the pDNA genome, we assumed an evolutionary rate of 1.52×10^−9^ s s^−1^ yr^−1^ for the plastid data set [79]. Ten years was used as an approximation for *g* (J. Q. Zhang, pers. obs.).

## Results

### Plastid sequence variation and haplotype distribution

**Figure 1:**
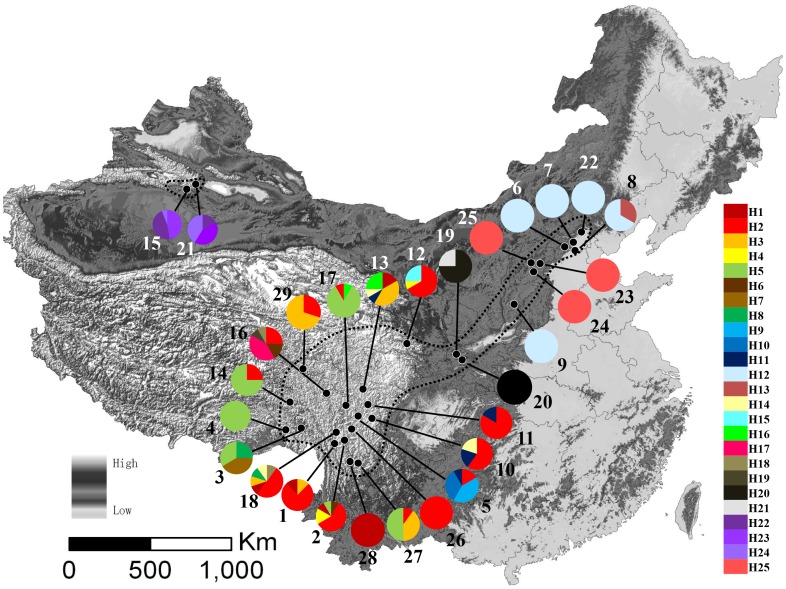
A map of the sampling sites and the geographic distribution of *Rhodiola kirilowii* haplotypes. Pie charts show the proportion of chlorotypes within each population. The numbers beside the circles represent population numbers listed in Table S1. Dash line on the map indicate the distribution area of *R. kirilowii*.

The length of aligned sequences was 941 bp for *trnL-F*, and 609 bp for *trnS-G*. After combining the two data sets, we detected 25 haplotypes (H1–H25) from the 306 sampled individuals of *R. kirilowii*, determined by 36 single nucleotide polymorphic sites and three indels. The length of these haplotypes varied between 1424 and 1491 bp. Eight haplotypes were unique to single populations, while the rest were shared by different populations (Table S2). Among these, H2 was the most common haplotype – occurring in 13 of the 29 populations – followed by H1, H3, H5, and H12 – occurring in five populations (Table S2). At the species level, the estimated haplotype diversity was *h* = 0.913. Haplotype diversity varied across populations, ranging from 0.000 to 0.788, with the LHX and QES populations having the highest *h* values (Table S1). Nucleotide diversity was *π* = 0.0036 at the species level, and it ranged from 0.000 to 0.0039, with population BS having the highest *π* value (Table S1). Within-population gene diversity (*H*_S_) was much lower than total gene diversity (*H*_T_) (0.367 and 0.914, respectively, Table 1). Of the 29 sampled populations, 11 harbored only a single haplotype, while the other 18 were polymorphic, with two or more haplotypes (Fig. 1; Table S1). Population SK harbored six haplotypes which was the most in any of the populations.

**Table 1:** Genetic diversity and genetic differentiation of 29 populations of *Rhodiola kirilowii* at the species level and group levels.

	Plastid DNA					ITS				
Groups	*H*_S_	*H*_T_	*π* (×10^−3^)	*G*_ST_	*N*_ST_	*H*_S_	*H*_T_	*π* (×10^−3^)	*G*_ST_	*N*_ST_
Total	0.367 (0.0597)	0.914 (0.0191)	3.563	0.599 (0.0637)	0.607 (0.0702)^ns^	0.156 (0.0457)	0.879 (0.0292)	10.996	0.822 (0.0509)	0.969 (0.0130)**
HM	0.489 (0.0701)	0.828 (0.0439)	2.818	0.409 (0.0805)	0.430 (0.1211)^ns^	0.089 (0.0500)	0.742 (0.0736)	3.829	0.880 (0.0645)	0.961 (0.0231)*
NC	0.302 (0.0867)	0.728 (0.0615)	1.207	0.586 (0.1311)	0.861 (0.0742)^ns^	0.302 (0.0867)	0.728 (0.0615)	3.365	0.586 (0.1311)	0.861 (0.0742)*
CA	-	-	1.940	-	-	-	-	0.000	-	-

HM, Hengduan Mountains; NC, northern China; CA, central Asia; * *N*_ST_ is significantly different from *G*_ST_ (*P*<0.05); **N_ST_ is significantly different from *G*_ST_ (*P*<0.01); ns, not significant.

### NrDNA ITS variation and ribotype distribution

The length of aligned ITS sequences was 618 bp. Twelve different ITS sequences (ribotypes) were recovered (R1–R12), three of which occurred in not more than one population. These ribotypes were determined by a total of 30 substitutions, and their length was in the range 610–618 bp. Among the ribotypes determined, only three occurred in a single population, while the other nine were shared by at least two populations (Table S3). The most widespread ribotypes were R7 and R8, both of which were found in eight populations (Table S3). Two populations in Xinjiang (WLMQ and NS) have the same ribotype, R11 (Table S1). Ribotype diversity was estimated to be *h* = 0.866 at the species level, ranging from 0.000 to 0.667 in different populations. Population DLS3 has the highest *h* value (Table S1). At the species level, nucleotide diversity was 0.010 (*π* = 0.010), but it varied across populations, ranging from 0.000 to 0.0016, with the LHS population having the highest *π* value (Table S1). Like the pDNA data, within-population gene diversity (*H*_S_) was much lower than total gene diversity (*H*_T_) (0.156 and 0.879, respectively; Table 1). Notably, 20 of the 29 populations only harbored a single ribotype, and only nine populations harbored more than one ribotype. Population LHS had the most ribotypes: three of them.

### Phylogenetic relationships and lineages divergence

**Figure 2:**
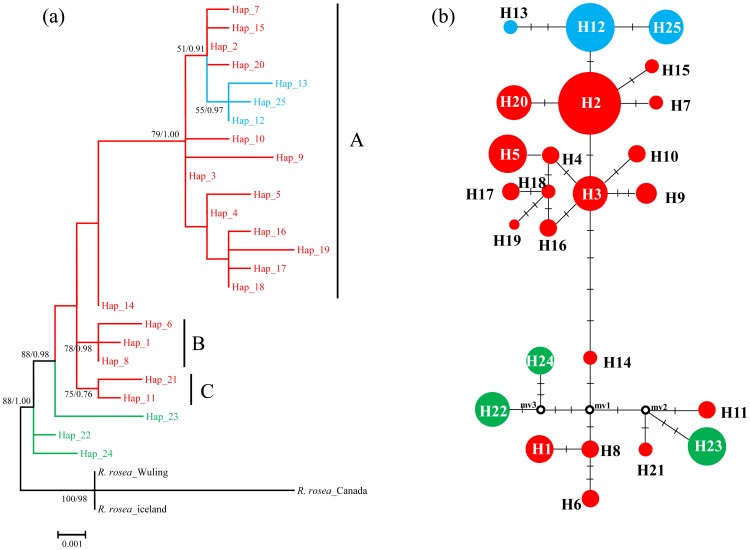
Phylogenetic relationships obtained by analysis of pDNA haplotypes. (a) Bayesian tree with numbers above the branches indicating bootstrap values greater than 50% for MP analysis and Bayesian posterior probabilities. (b) NETWORK-derived genealogical relationship. The sizes of the circles in the network are proportional to the observed frequencies of the haplotypes. The small black bars represent mutation steps and the red dots represent missing chlorotypes. For both subfigures, different colors represent different geographic origins of haplotypes: red, the Qinghai-Tibetan Plateau and Hengduan Mountains; blue, northern China; green, central Asia.

For the plastid data, our phylogenetic analyses based on MP and Bayesian methods yielded trees with largely congruent topologies (Fig. 2a). The monophyly of *R. kirilowii* was well supported. The 25 haplotypes clustered into three multiple-haplotype clades (A, B, and C) (Fig. 2a) and four monotypic ones. The haplotype network (Fig. 2b) depicted relationships between haplotypes in more detail. The three haplotypes (H22, 23, 24) in the central Asian populations (WLMQ and NS) resided at the base of the phylogeny. Three haplotypes found in northern China were nested in clade A, which comprised both the HM and northern China haplotypes.

**Figure 3:**
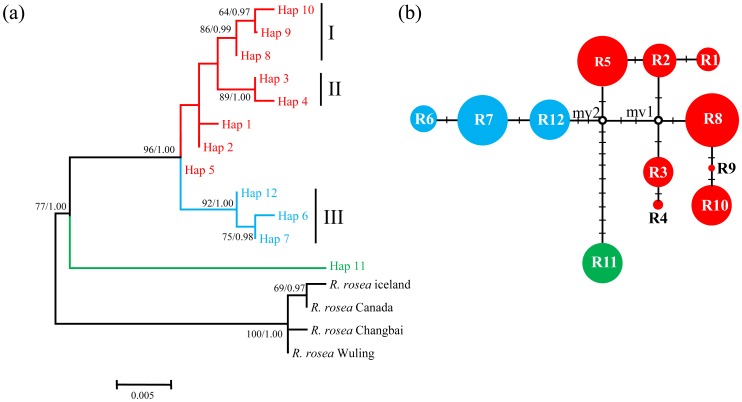
Phylogenetic relationships obtained by analysis of ITS ribotypes. (a) Bayesian tree with numbers above the branches indicating bootstrap values greater than 50% for MP analysis and Bayesian posterior probabilities. (b) NETWORK-derived genealogical relationship. The sizes of the circles in the network are proportional to the observed frequencies of the ribotypes. The small black bars represent mutation steps and the red dots represent missing ribotypes. For both subfigures, different colors represent different geographic origins of ribotypes: red, the Qinghai-Tibetan Plateau and Hengduan Mountains; blue, northern China; green, central Asia.

**Figure 4:**
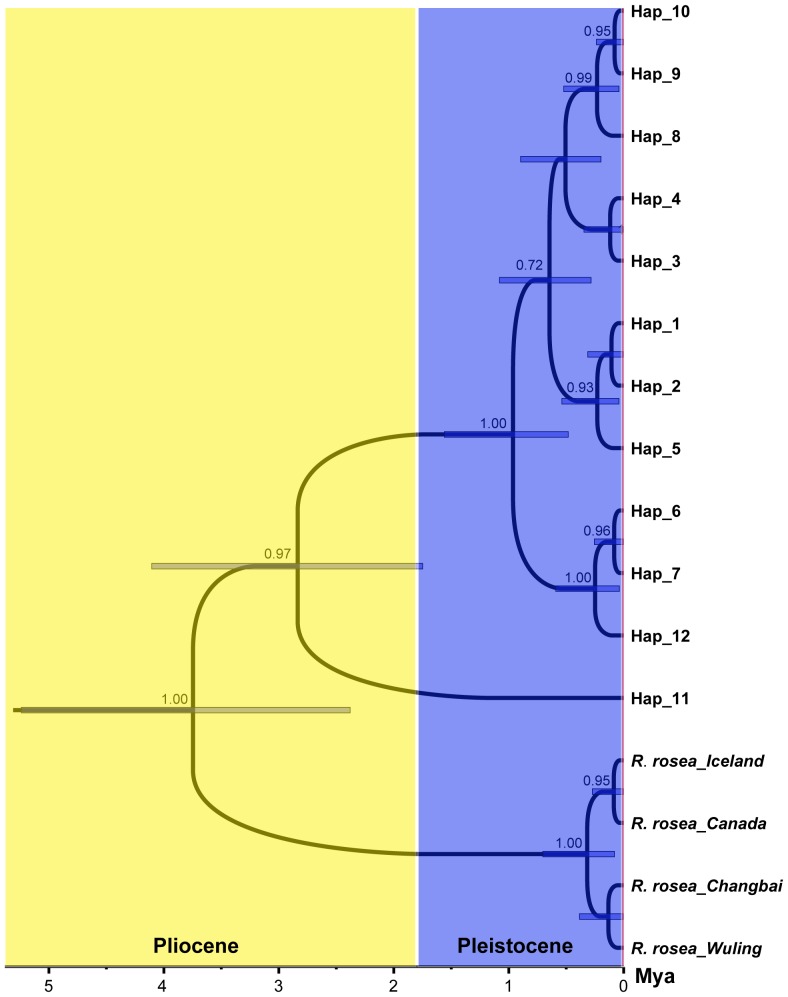
Maximum clade credibility tree of the ribotypes generated from BEAST. Gray bars indicate 95% highest posterior density intervals.

For the ITS data, phylogenetic relationships reconstructed by Bayesian and MP methods were consistent (Fig. 3a). All 12 ribotypes formed a well-supported clade. Ribotype 11 from central Asia was highly diverged from the other ribotypes, and the other 11 ribotypes formed a well-support clade (BP  = 100, PP  = 1.00); several major clades with high bootstrap values and posterior probabilities were recognized (I, II, and III). The ribotype network (Fig. 3b) contained the same relationships as the phylogenetic trees (Fig. 3a), although the ribotype relationships were shown with more details. The dating analysis showed that Ribotype 11 diverged from the other ribotypes at 2.84 Mya (95% HPD: 1.75–4.10 Mya), i.e. in the late Pliocene (Fig. 4). Further diversification of the multiple-ribotype clade took place at 0.96 Mya (95% HPD: 0.48–1.56 Mya), during the Pleistocene.

### Population structure

Population differentiation of *R. kirilowii* was particularly high, with *G*_ST_ values of 0.599 and 0.822 for pDNA and ITS data, respectively (Table 1). A significantly higher value of *N*_ST_ than *G*_ST_ was detected by the permutation tests examining the ITS data (*P*<0.01), indicating a strong phylogeographic structure in *R. kirilowii*. In contrast, the permutation tests of pDNA showed that *N*_ST_ was not significantly higher than *G*_ST_ (Table 1). In the SAMOVA analysis for both pDNA and ITS data, the *F*_CT_ value increased progressively as the value of *K* increased from two to ten (Fig. S1), making identifying the number *K* of groups ambiguous. However, the phylogeny based on both pDNA and ITS data sets showed consistency between the three geographic regions (i.e. the Hengduan Mountains, northern China, and central Asia) and lineage divergence. Thus, we divided the 29 populations collected into three geographic groups as mentioned above. Pairwise *F*_ST_ between the three geographic regions varied from 0.5387 to 0.9163, and from 0.1937 to 0.5768 for pDNA and ITS data, respectively (Table 2), suggesting high genetic differentiation between the regions. The Mantel test based on both pDNA and ITS data revealed a significant pattern of isolation-by-distance (pDNA: *r* = 0.452, *P*<0.01; ITS: *r* = 0.489, *P*<0.01).

**Table 2:** Pairwise comparisons of *F*_ST_ among regions estimated from internal transcribed spacer (ITS) sequences (upper part) and pDNA sequences (lower part) of *Rhodiola kirilowii*.

	HM	Northern China	Central Asia
Hengduan Mountains (HM)		0.1937	0.3404
Northern China	0.5387		0.5768
Central Asia	0.8878	0.9163	

All values are significant at the 0.01 level in a permutation tests (1000 permutations).

### Demographic analyses

Mismatch distribution analysis suggested that, under a model of population expansion, only Clade A of the pDNA tree, which included most haplotypes of the QTP and all haplotypes of northern China, showed a unimodal mismatch distribution (Fig. S2). The observed variance (*SSD*) and the raggedness index for Clade A were not significantly different from the expectation under the population expansion model (Table 4). In addition, Tajima's *D* and Fu's *F*s were also significantly negative (Table 4). Thus, there is a strong indication that Clade A underwent a rapid expansion. The estimated time of this expansion was 0.168 Mya (Table 4).

## Discussion

### Pliocene intraspecific divergence, Pleistocene range expansions, and glacial refugia of *R. kirilowii*

Both our phylogenetic and NETWORK analysis showed that the central Asia populations (Population WLMQ and NS) are clearly divergent from the other populations of *R. kirilowii*. The results of BEAST analysis based on ITS data showed that this divergence took place in the middle to late Pliocene (Fig. 4), during which the most recent uplift of the Qinghai-Tibetan Plateau occurred [80]. Although the uplift of the QTP began around 40 Mya [81]–[84], the recent intensive uplift took place 3.4 to 1.6 Mya and resulted in the QTP reaching its present elevation, i.e. more than 4,000 m [80]. Meanwhile, the recent uplift of the QTP created the Asian monsoon climate, which resulted in the rapid expansion of dry habitats on the platform of the QTP and in the interior of Asia [85]. *Rhodiola kirilowii* often grows at forest margins, on grassy slopes, often in partial shade near mountain peaks [51]. The vegetation of the QTP before its last intensive uplift was mostly composed of deciduous broadleaved forest and coniferous mixed forest [86], [87], habitats where *R. kirilowii* usually occurs. Thus, *R. kirilowii* could have been widely spread from central Asia (i.e. Tianshan Mountain) to the QTP area. However, after the recent intensive uplifts, the flora of the northern and western part of the QTP was replaced by alpine scrub grasslands and desert [88] due to its drier and colder environment. Thus, the distribution of *R. kirilowii* was fragmented by the intensive uplift of the QTP in the late Pliocene. Pliocene uplift of the QTP has been suggested as a driving force for intraspecific divergence of several alpine species in this region [33], [34], [41], [42], and most recently for two conifer species, *Taxus wallichiana* Zucc. [89] and *Picea likiangensis* (Franch.) E. Pritz. [13]. However, most of the previous studies revealed divergence between eastern Himalaya and Hengduan Mountains [13], [89]; in contrast, our results demonstrate a rare divergence between central Asia and the Hengduan Mountains area. Numerous studies on other shrubs, herbs and animal groups with dated molecular phylogenies have also indicated extensive species diversification in the QTP and adjacent area within the Pliocene [15], [23], [32]. Therefore, uplifts of the QTP caused species' distribution fragmentation, which promoted both intraspecific and interspecific divergence on a large scale in this region.

The HM area has long been recognized not only as an important species center for Tertiary elements, but also a principal glacial refugium for many plants in East Asia [26], [29], [31], [41]. A glacial refugium is characterized by high genetic diversity and haplotype uniqueness [90]. These features were clearly revealed in the populations of the HM area, especially populations BM, DF, and LHX (Fig. 1; Table S1). All three of these populations are characterized by high genetic diversity and haplotype uniqueness. Thus, our results confirm the HM area as an important Quaternary glacial refugium, as documented in previous studies [26], [29], [31], [34], [41], [91], [92]. The populations of central Asia diverged in the Pliocene as discussed above, thus they may have survived the Last Glacial Maximum (LGM) in the East Tianshan Mountains, which other studies have also identified as a potential major refugium for plants in northwest China [93].

Few phylogeographic studies have discussed the floristic relationship of HM with the vast adjacent area in northern China, which is dominated by homogeneous grassland [44]. With *R. kirilowii*'s distribution extending from the HM to the mountains of northern China, we have the chance to discuss this issue. Both our pDNA and ITS results show that most populations from northern China harbor only one haplotype or ribotype (Fig. 1). Only three closely related haplotypes (H12, H13, and H 25) and ribotypes (R6, R7, and R12) were found in the northern China groups. In contrast, most populations from the HM area harbored more than one haplotype or ribotype. On the regional level, the total gene diversity of the HM was also higher than that of northern China (Table 1). Furthermore, our phylogenetic analysis showed that the three haplotypes and ribotypes of the northern China group were nested within a large clade which includes most of the HM haplotypes (Figs. 2 & 3). All the above evidence indicates that the northern China populations originated from ones in the HM as a result of population expansion. Our mismatch distribution analysis revealed a unimodal pattern for Clade A of the pDNA phylogenetic tree (Fig. S2), indicating a northeastward range expansion. Furthermore, negative values of Fu's *F*_S_ and Tajima's *D* were also observed, which again reinforce the expansion scenario (Table 4). No such range expansions were indicated for the other two clades (Table 4). Using a mean pDNA mutation rate of 1.52×10^−9^ substitutions per site per year [79], the range expansion of Clade A was estimated to have occurred 168 kya, which fell within the Guxiang (the Penultimate) glacial period of the QTP and the HM area. During the glacial period, the cold-adapted *R. kirilowii* could respond to these oscillations by moving down the mountains and migrating along the mountain ridges of northern China, e.g., the Qingling Mountains, the Taihang Mountains and the Lvliang Mountains. When the temperature increased after the glacial period, this species retreated into the habitats on mountain tops. The distribution pattern exhibited by *R. kirilowii* is similar to that of *Stellera chamaejasme* L., a perennial herb mainly occurring in the HM and northern China [42]. The present distribution of this species in northern China resulted from a rapid post-glaciation expansion from the HM [42]. However, another species with a similar distribution pattern displayed a different pattern: the two highly divergent lineages corresponding to the eastern QTP and away from the QTP (northern China) of *Incarvillea sinensis* diverged 4.4 Mya, which was much earlier than *R. kirilowii* and *S. chamaejasme* [38].

### Population structure and genetic diversity of naturally isolated populations

For both pDNA and ITS data sets, we detected a significant high total genetic diversity (pDNA, *H*_T_0.914, ITS, *H*_T_ = 0.879; Table 1) based on 29 populations and 306 individuals of *R. kirilowii*. Meanwhile, the estimate of genetic differentiation among the 29 populations was also high based on both pDNA and ITS data (*F*_ST_ = 0.598 and 0.826, separately). A high total genetic diversity has also been documented in two previous phylogeographic studies involving two other congeneric species (i.e. *R. alsia* (Fröd.) S. H. Fu and *R. dumulosa* (Franch.) S. H. Fu; *H*_T_ = 0.950 and 0.981, respectively) [91], [94]. However, the total genetic diversities of these three species are all higher than other alpine species found in the QTP and adjacent area, for example, *Pedicularis longiflora* Rudolph (*H*_T_ = 0.770) [31], *Aconitum gymnandrum* Maxim. (*H*_T_ = 0.739) [34], *Incarvillea sinensis* Lam. (*H*_T_ = 0.677) [38], and *Taxus wallichiana* Zucc. (*H*_T_ = 0.809) [89]. Thus, in the case of *R. kirilowii*, the hypothesis that genetic variability decreased as a result of population fragmentation was not supported. Although numerous studies have revealed that habitat fragmentation can reduce genetic diversity due to restricted gene flow, inbreeding and genetic drift in isolated small populations [47], some studies have told another story, namely that overall genetic diversity can be maintained or even increased by fragmentation, as a result of allopatric speciation occurring due to isolation [15], [48], [50], [95]. *Rhodiola kirilowii* grows exclusively at forest margins, on grassy slopes near mountain peaks at an elevation of 2,000 to 5,600 m, effectively occupying ‘ecological islands’ which are naturally fragmented. These conditions are found especially in the HM area, where severe uplift created high mountains and deeps valleys, providing great opportunities for species like *R. krilowii* to undergo allopatric divergence. A high genetic differentiation among populations could be the result of spatially isolated populations with restricted gene flow [96], [97]. The scenario of high genetic diversity caused by isolation was also supported by the Mantel test between the pairwise *F*_ST_ and geographic distance (for pDNA, *r* = 0.452, *P* = 0.0000; for ITS, *r* = 0.489, *P* = 0.0000), indicating a significant and strong isolation-by-distance pattern.

Compared to the high total genetic diversity as mentioned above, the mean within-population genetic diversity at both species and regional level was low (Table 1). The AMOVA analysis results also suggested that most genetic variations should be attributed to among-region or among-population levels for the ITS data (Table 3). Nevertheless, the AMOVA analysis of the pDNA data revealed that within-population variation is greater than among-population variation (Table 3). The pattern was confirmed by the results of the permutation test, which revealed a significantly higher *N*_ST_ value than *G*_ST_ value in the ITS data but not in the pDNA data (Table 1). This phenomenon could be ascribed to repeated population expansion and contraction responding to climate oscillations during the Quaternary, especially in the HM area. *Rhodiola kirilowii* is a cold-adapted species, so when temperatures rose during the inter-glacial period, populations of this species were able to retreat to mountain peaks, which served as refuges. Such refuges may harbor high genetic diversity, as indicated in this study. However, this high genetic diversity was not revealed in the ITS data set, which suggests frequent gene flow via pollen within populations and between close populations. More strikingly, all individuals from central Asia (populations WLMQ and NS; Table S1) all bear the same ribotype. On the other hand, these populations harbor three pDNA haplotypes. Our results indicated that in *R. kirilowii*, gene flow via pollen can be very efficient in closely distributed populations, while the dispersal ability of seeds is limited. Once the capsules of *R. kirilowii* split, the seeds are ejected and dispersed by wind. This kind of dispersal mechanism could be easily hampered by deep valleys and high mountains between populations. At a larger scale, the AMOVA analysis showed that the variation at the regional level accounted for 79.26% and 45.28% of the ITS and pDNA data, respectively (Table 3). Pairwise *F*_ST_ among the three geographic regions also suggested a high level of divergence, which may be the result of the long isolation of these three regions.

**Table 3:** Analysis of molecular variance (amova) of chlorotypes and ITS ribotypes for *Rhodiola kirilowii* populations, partitioned by subspecies and regions, respectively.

		ITS				pDNA			
Source of variation	df	SS	VC	PV (%)	*F* statistics	SS	VC	PV (%)	*F* statistics
Among regions	2	747.86	3.79	79.26	*F*_SC_ = 0.91793*	274.98	1.33	45.28	*F*_SC_ = 0.41521*
Among populations	26	268.82	0.91	19.03	*F*_ST_ = 0.98298*	220.79	0.67	22.72	*F*_ST_ = 0.67998*
Within populations	304	24.75	0.08	1.70	*F*_CT_ = 0.79265*	286.90	0.94	32.00	*F*_CT_ = 0.45277*
Total	332	1041.42	4.78	-	-	782.67	2.95	-	-

df, degrees of freedom; SS, sum of squares; VC, variance components; PV, percentage of variation. *F*_SC_, correlation within populations relative to group; *F*_ST_, correlation within populations relative to total; *F*_CT_, correlation within groups relative to total. *, *P*<0.001, 1000 permutations.

**Table 4:** Results of the mismatch distribution analysis and neutrality tests of the three multiple-haplotype pDNA clades.

Haplotype group	*τ*	t (Mya)	*SSD*	p-value	Raggedness index	p-value	Tajima's *D*	p-value	*F*_S_	p-value
Clade A	0.793	0.168	0.005	0.390	0.029	0.630	−0.819	0.041	−2.23	0.047
Clade B	4.223	-	0.102	0.08	0.388	0.330	1.361	0.916	2.968	0.924
Clade C	8.225	-	0.386	0.030	0.837	0.010	1.982	0.988	5.342	0.987
Total	11.021	-	0.015	0.75	0.029	0.410	−0.071	0.281	8.393	0.926

## Supporting Information

Figure S1 **Correlation between the** ***F*** **statistics and grouping number (** ***K*** **= 2–10) from the SAMOVA results.** (a) results based on pDNA haplotypes; (b) results based on ITS ribotypes.
(TIF)

Figure S2 **Mismatch distribution analyses of the three multiple-haplotype pDNA clades.** The histogram of observed mismatch frequencies and the best-fit curve of the sudden expansion model was shown.
(TIF)

Table S1 **Locations of populations of** ***R. kirilowii*** **sampled, sample sizes (n), frequencies of cpDNA haplotypes and ITS sequences per population, and estimates of haplotype diversity and nucleotide diversity for chlorotypes and ribotypes within populations.**
(DOCX)

Table S2 **Haplotype composition of 29 sampled populations of** ***Rhodiola kirilowii*** **.**
(DOCX)

Table S3 **Ribotypes composition of 29 sampled populations of** ***Rhodiola kirilowii*** **.**
(DOCX)
