# Supplementary figures and images for: Phylogeography of Rhodiola kirilowii (Crassulaceae): A Story of Miocene Divergence and Quaternary Expansion
Source: PLoS One. 2014 Nov 12;9(11):e112923. doi: 10.1371/journal.pone.0112923 (PMC4229298; doi:10.1371/journal.pone.0112923)

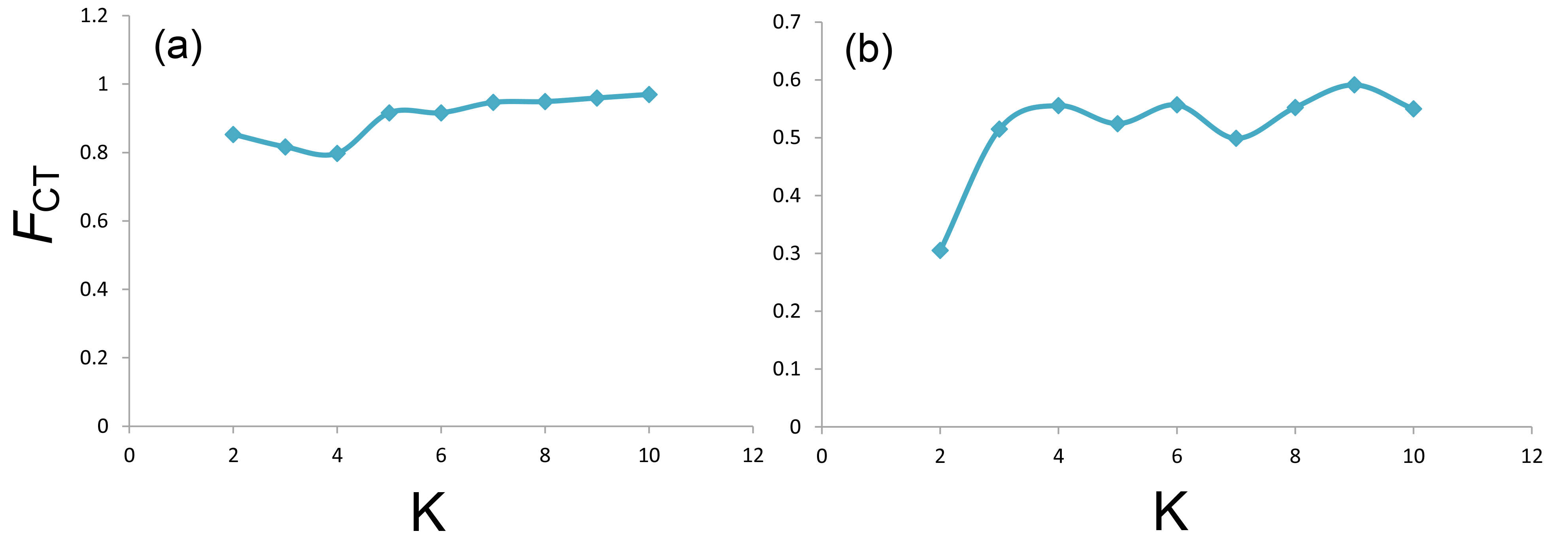

Supplement: Figure S1 — Correlation between the F statistics and grouping number ( K = 2–10) from the SAMOVA results. (a) results based on pDNA haplotypes; (b) results based on ITS ribotypes. (TIF) [file pone.0112923.s001.tif]

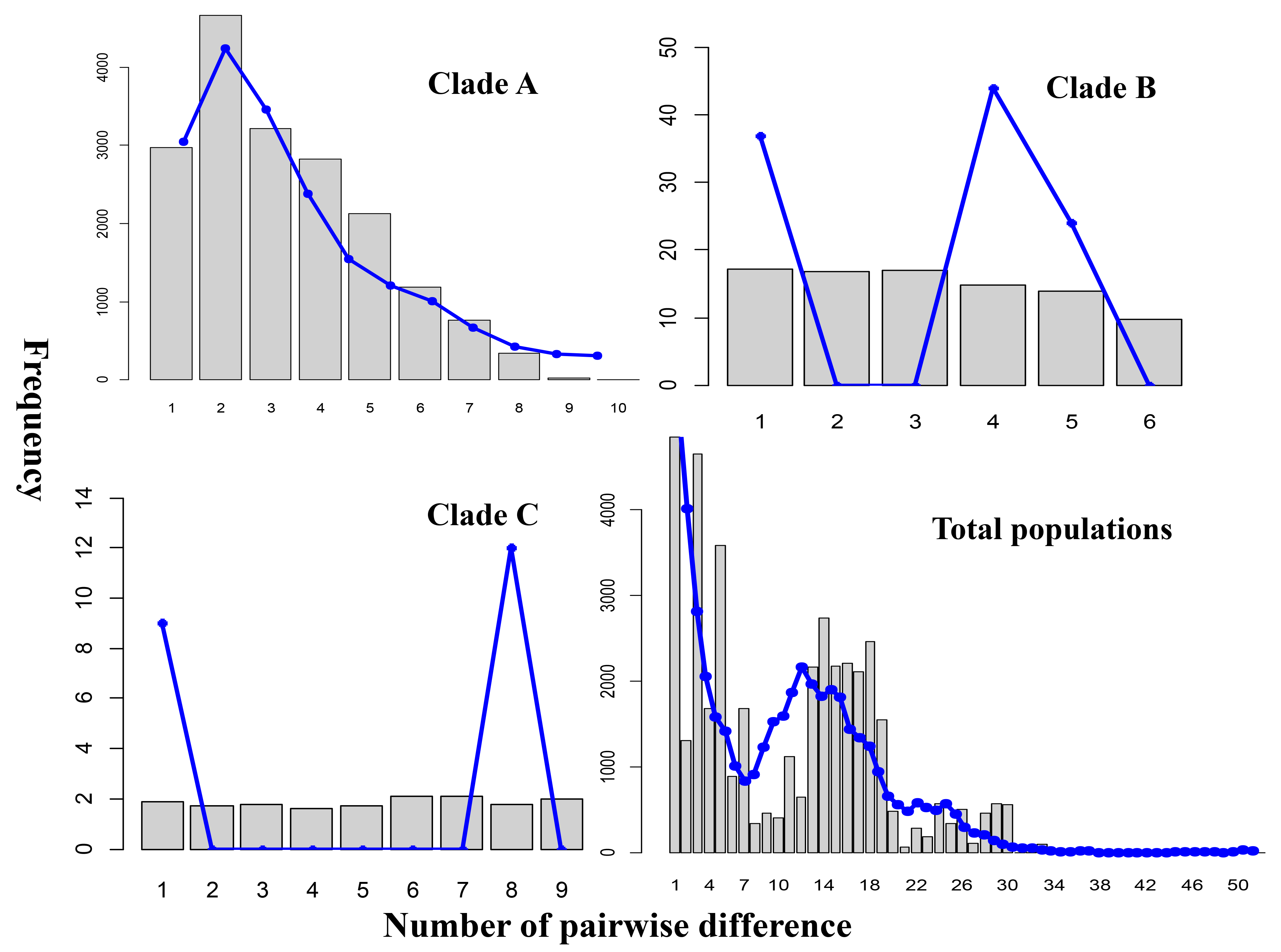

Supplement: Figure S2 — Mismatch distribution analyses of the three multiple-haplotype pDNA clades. The histogram of observed mismatch frequencies and the best-fit curve of the sudden expansion model was shown. (TIF) [file pone.0112923.s002.tif]
