# Supplementary material for: Phylogeography of Rhodiola kirilowii (Crassulaceae): A Story of Miocene Divergence and Quaternary Expansion
Source: PLoS One. 2014 Nov 12;9(11):e112923. doi: 10.1371/journal.pone.0112923 (PMC4229298; doi:10.1371/journal.pone.0112923)
Supplement: Table S1 — Locations of populations of R. kirilowii sampled, sample sizes (n), frequencies of cpDNA haplotypes and ITS sequences per population, and estimates of haplotype diversity and nucleotide diversity for chlorotypes and ribotypes within populations. (DOCX) [file pone.0112923.s003.docx]

**Table S1.** Locations of populations of *R. kirilowii* sampled, sample sizes (n), frequencies of cpDNA haplotypes and ITS sequences per population, and estimates of haplotype diversity and nucleotide diversity for chlorotypes and ribotypes within populations.

|  |  |  |  |  |  |  | Chlorotypes | | | Ribotypes | | |
| --- | --- | --- | --- | --- | --- | --- | --- | --- | --- | --- | --- | --- |
| Population | Abbr. | Locality | Lat. (N) | Long. (E) | Alt. (m) | N | Haplotypes nos. | *h* (SD) | *π* (SD) in ‰ | ITS sequences nos. | *h* (SD) | *π* (SD) in ‰ |
| 1 | ADZ | Baima Mt. YN | 28° 20.01´ | 99° 04.38´ | 4300 | 8 | H1(1) H2(6) H3(1) | 0.464 (0.04) | 1.707 (1.109) | R1 (8) | 0 | 0 |
| 2 | BM | Deqin YN | 28° 23.06´ | 99° 00.34´ | 4300 | 12 | H1(1) H2(7) H4(2) H5(1) H6(1) | 0.667 (0.02) | 2.656 (0.89) | R2 (12) | 0 | 0 |
| 3 | BS | Basu XZ | 29° 19.25´ | 97° 02.58´ | 4800 | 12 | H5(3) H7(5) H8(4) | 0.75 (0.01) | 3.937 (0.55) | R3 (12) | 0 | 0 |
| 4 | CY | Chayu XZ | 28° 35.41 ´ | 97° 57.20´ | 3968 | 8 | H5 (8) | 0 | 0 | R3 (4) R4 (4) | 0.6 (0.017) | 0.977 (0.21) |
| 5 | DF | Daofu SC | 31° 01.33´ | 101° 14.20´ | 4100 | 12 | H2(2) H9(5) H10(4) H11(1) | 0.742 (0.007) | 3.346 (0.65) | R5 (12) | 0 | 0 |
| 6 | DLS1 | Dongling Mt. BJ | 39° 59.27´ | 115° 25.36´ | 1825 | 12 | H12(12) | 0 | 0 | R6 (7) R7 (5) | 0.53 (0.006) | 0.861 (0.12) |
| 7 | DLS2 | Xiaolongmen BJ | 39° 59.21´ | 115° 25.38´ | 2303 | 12 | H12(12) | 0 | 0 | R6 (4) R7 (8) | 0.485 (0.011) | 0.787 (0.17) |
| 8 | DLS3 | Mentougou BJ | 39° 59.34´ | 115° 25.28´ | 2290 | 9 | H12(3) H13 (6) | 0.667 (0.09) | 0.932 (0.44) | R6 (3) R7 (6) | 0.667 (0.098) | 1.082 (0.51) |
| 9 | GDS | Guandi Mt. SX | 37° 52.37´ | 111° 26.05´ | 2100 | 12 | H12(12) | 0 | 0 | R7 (12) | 0 | 0 |
| 10 | HS | Heishui SC | 32° 13.60´ | 102° 35.06´ | 4300 | 10 | H2(6) H11(2) H14(2) | 0.7 (0.048) | 2.952 (0.94) | R8 (10) | 0 | 0 |
| 11 | JC | Jinchuan SC | 31° 25.39´ | 102° 05.04´ | 3000 | 11 | H2(10) H11(2) | 0.182 (0.020) | 1.022 (0.81) | R8 (12) | 0 | 0 |
| 12 | LHS | Kangle GS | 34° 56.29´ | 103° 45.23´ | 2900 | 12 | H2(8) H4(1) H15(3) | 0.53 (0.018) | 0.522 (0.20) | R8 (7) R9 (1) R10 (4) | 0.591 (0.012) | 1.648 (0.28) |
| 13 | LHX | Luhuo SC | 31° 45.72´ | 100° 45.45´ | 4000 | 12 | H1(2) H3(5) H11(1) H14(1) H16(3) | 0.788 (0.008) | 2.622 (0.59) | R8 (12) | 0 | 0 |
| 14 | LWQ | Leiwuqi XZ | 31°31.07 ´ | 96° 21.27 ´ | 4070 | 8 | H2(2) H5(6) | 0.5 (0.070) | 1.399 (0.74) | R8 (8) | 0 | 0 |
| 15 | NS | Urumqi XJ | 43° 28.31´ | 87° 00.41´ | 1889 | 17 | H22(8) H23(8) H24(1) | 0.523 (0.002) | 2.561 (0.23) | R11 (17) | 0 | 0 |
| 16 | QES | Dege SC | 31° 48.80´ | 98° 34.80´ | 4800 | 12 | H2(3) H6(2) H17(5) H18(1) H19(1) | 0.788 (0.008) | 3.371 (0.78) | R8 (12) | 0 | 0 |
| 17 | SJS | Maerkang SC | 31° 51.81´ | 101° 20.30´ | 3200 | 12 | H2 (1) H5(10) H16(1) | 0.318 (0.027) | 0.87 (0.44) | R8 (12) | 0 | 0 |
| 18 | SK | Shika Mt. YN | 27° 53.06´ | 99° 33.45´ | 3900 | 10 | H1(1) H2(5) H3(1) H8(1) H14(1) H18(1) | 0.778 (0.019) | 2.873 (0.70) | R5 (10) | 0 | 0 |
| 19 | TBS1 | Taibai Mt. SN | 34° 01.12´ | 107° 51.38´ | 2900 | 12 | H20(9) H21(3) | 0.409 (0.018) | 3.45 (1.12) | R10 (12) | 0 | 0 |
| 20 | TBS2 | Taibai Mt. SN | 33° 59.10´ | 107° 47.33´ | 3528 | 12 | H20(12) | 0 | 0 | R10 (12) | 0 | 0 |
| 21 | WLMQ | Urumqi XJ | 43° 07.31 ´ | 87° 04.21 ´ | 2550 | 17 | H22(3) H23(7) H24(7) | 0.527 (0.004) | 2.582 (0.31) | R11 (17) | 0 | 0 |
| 22 | WLS | Wuling Mt. BJ | 40° 35.97´ | 117° 29.10´ | 2057 | 16 | H12(16) | 0 | 0 | R7 (16) | 0 | 0 |
| 23 | WT1 | Wutai Mt. SX | 39° 03.50´ | 113° 38.99´ | 2500 | 12 | H25(12) | 0 | 0 | R7 (4) R12 (8) | 0.513 (0.007) | 0.833 (0.13) |
| 24 | WT2 | Wutai Mt. SX | 39° 01.08´ | 113° 33.94´ | 1900 | 12 | H25(12) | 0.167 (0.018) | 0.466 (0.38) | R7 (6) R12 (6) | 0.533 (0.009) | 0.866 (0.15) |
| 25 | WT3 | Wutai Mt. SX | 39° 01.58´ | 113° 31.45´ | 2300 | 12 | H25(12) | 0 | 0 | R7 (2) R12 (10) | 0.303 (0.022) | 0.492 (0.24) |
| 26 | XC | Xiangcheng SC | 29° 09.27´ | 99° 56.01´ | 4500 | 12 | H2(12) | 0 | 0 | R2 (3) R5 (9) | 0.327 (0.023) | 0.53 (0.25) |
| 27 | YL1 | Lijinag YN | 27° 02.73´ | 100° 11.68´ | 3700 | 10 | H2(1) H3(4) H5(5) | 0.644 (0.010) | 1.304 (0.19) | R5 (10) | 0 | 0 |
| 28 | YL2 | Yulong Mt. YN | 27° 01.75´ | 100° 11.34´ | 3400 | 6 | H1(6) | 0 | 0 | R5 (6) | 0 | 0 |
| 29 | YS | Yushu QH | 32° 51.19´ | 97° 08.73´ | 4050 | 10 | H2(3) H3(7) | 0.467 (0.017) | 0.328 (0.09) | R8 (10) | 0 | 0 |

Abbreviation: BJ, Beijing; GS, Gansu; SC, Sichuan; SN, Shaanxi; SX, Shanxi; XJ, Xinjiang; XZ, Xizang; YN, Yunnan.
