# Supplementary material for: Phylogeography of Rhodiola kirilowii (Crassulaceae): A Story of Miocene Divergence and Quaternary Expansion
Source: PLoS One. 2014 Nov 12;9(11):e112923. doi: 10.1371/journal.pone.0112923 (PMC4229298; doi:10.1371/journal.pone.0112923)
Supplement: Table S3 — Ribotypes composition of 29 sampled populations of Rhodiola kirilowii . (DOCX) [file pone.0112923.s005.docx]

**Table S3.** Ribotypes composition of 29 sampled populations of *Rhodiola kirilowii.*

|  |  | Ribotypes compositions | | | | | | | | | | | |
| --- | --- | --- | --- | --- | --- | --- | --- | --- | --- | --- | --- | --- | --- |
| Population | Total | 1 | 2 | 3 | 4 | 5 | 6 | 7 | 8 | 9 | 10 | 11 | 12 |
| CY | 8 |  |  | 4 | 4 |  |  |  |  |  |  |  |  |
| WLMQ | 17 |  |  |  |  |  |  |  |  |  |  | 17 |  |
| NS | 17 |  |  |  |  |  |  |  |  |  |  | 17 |  |
| XC | 12 |  | 3 |  |  | 9 |  |  |  |  |  |  |  |
| BS | 12 |  |  | 12 |  |  |  |  |  |  |  |  |  |
| BM | 12 |  | 12 |  |  |  |  |  |  |  |  |  |  |
| LWQ | 8 |  |  |  |  |  |  |  | 8 |  |  |  |  |
| DLS3 | 9 |  |  |  |  |  | 3 | 6 |  |  |  |  |  |
| YS | 10 |  |  |  |  |  |  |  | 10 |  |  |  |  |
| YL1 | 10 |  |  |  |  | 10 |  |  |  |  |  |  |  |
| YL2 | 6 |  |  |  |  | 6 |  |  |  |  |  |  |  |
| SK | 10 |  |  |  |  | 10 |  |  |  |  |  |  |  |
| ADZ | 8 | 8 |  |  |  |  |  |  |  |  |  |  |  |
| WLS | 16 |  |  |  |  |  |  | 16 |  |  |  |  |  |
| DLS1 | 12 |  |  |  |  |  | 7 | 5 |  |  |  |  |  |
| LHS | 12 |  |  |  |  |  |  |  | 7 | 1 | 4 |  |  |
| SJS | 12 |  |  |  |  |  |  |  | 12 |  |  |  |  |
| JC | 12 |  |  |  |  |  |  |  | 12 |  |  |  |  |
| HS | 10 |  |  |  |  |  |  |  | 10 |  |  |  |  |
| LHX | 12 |  |  |  |  |  |  |  | 12 |  |  |  |  |
| DLS2 | 12 |  |  |  |  |  | 4 | 8 |  |  |  |  |  |
| DF | 12 |  |  |  |  | 12 |  |  |  |  |  |  |  |
| QES | 12 |  |  |  |  |  |  |  | 12 |  |  |  |  |
| WT1 | 12 |  |  |  |  |  |  | 4 |  |  |  |  | 8 |
| GDS | 12 |  |  |  |  |  |  | 12 |  |  |  |  |  |
| WT2 | 12 |  |  |  |  |  |  | 6 |  |  |  |  | 6 |
| WT3 | 12 |  |  |  |  |  |  | 2 |  |  |  |  | 10 |
| TBS1 | 12 |  |  |  |  |  |  |  |  |  | 12 |  |  |
| TBS2 | 12 |  |  |  |  |  |  |  |  |  | 12 |  |  |
